# **A new species of *Parapleurocryptella*** **Bourdon, 1972 (Isopoda: Bopyridae) from deep water squat lobster hosts of New Zealand with description of larval stages and transfer of the genus to Pleurocryptellinae**

**DOI:** 10.1007/s11230-025-10255-7

**Published:** 2025-11-04

**Authors:** Grace Kokasko, Jason D. Williams, Christopher B. Boyko, Kareen E. Schnabel

**Affiliations:** 1 Department of Biology and Marine Biology, University of North Carolina Wilmington, Wilmington, NC 28403 USA; 2 Department of Biology, Hofstra University, Hempstead, NY 11549 USA; 3 Division of Invertebrate Zoology, American Museum of Natural History, New York, NY 10024 USA; 4 Marine Biodiversity & Biosecurity, New Zealand Institute for Earth Science Ltd, Kilbirnie, Private Bag 14901, Wellington, New Zealand

## Abstract

Squat lobsters (Decapoda: Anomura) harbor a high diversity of symbionts, including many parasitic isopod species (Epicaridea: Bopyridae) that reside in their branchial chambers. We describe the bopyrid *Parapleurocryptella poha ***n. sp.,** found infesting at least four species of squat lobsters from New Zealand. The new species displays morphological traits characteristic of the genus *Parapleurocryptella* Bourdon, 1972 but it is distinct from the two other described species, *P. minuta* Bourdon, 1972 and *P. elasmonoti* Bourdon, 1972, based on the form of the female head as well as male antennal segmentation, midventral tubercles, and dactyl size. Additionally, the new species is widely separated geographically and occurs on different host species compared to others in the genus. The morphology of mature male and female specimens is described, and we show a significant positive correlation of mature female parasite length with host size. The first description of the epicaridium larva of any species of *Parapleurocryptella* is provided and illustrated using SEM. Epicaridium larvae of the new species possess posterior external yolk sacs, a feature previously reported for larvae in only two other bopyrid genera: *Pleurocryptella* Bonnier, 1900 and *Paragigantione* Barnard, 1920. A discussion of the potential impacts of this structure to our understanding of bopyrid life cycles is provided. Additionally, the genera *Pleurocryptina* Nierstrasz & Brender à Brandis, 1929 and *Pagurocryptella* Boyko & Williams, 2010 are transferred to Pleurocryptellinae from Pseudioninae and *Pseudione kossmanni* is transferred to *Pleurocryptella* as *Pleurocryptella kossmanni* (Nierstrasz and Brender à Brendis, 1923) **nov. comb.** based on examination of the holotype.

## Introduction

Squat lobsters (Decapoda: Anomura) are crustaceans with a lobster-like morphology but which are compressed along a dorsoventral plane (Baba et al., 2008). Despite their common name, they are more closely related to hermit crabs, stone crabs, and mole and sand crabs (Schnabel et al., 2011a). Although superficially morphologically similar, squat lobsters are not a monophyletic grouping and the families are placed within two superfamilies: Chirostyloidea and Galatheoidea (Schnabel et al., 2011a; Dong et al., 2021). Galatheoidea is the more biodiverse superfamily and is made up of seven families including Porcellanidae (porcelain crabs that show a carcinized rather than lobster-like morphology), three families of fossil taxa, and three families of recent squat lobsters that contain almost 1100 described species (DecaNet, 2025). The Indo-Australian Archipelago is a hotspot for squat lobster diversity (Schnabel et al., 2011b), notably off the coast of New Zealand where the samples for this study were collected. The waters around New Zealand contain over 150 known species of squat lobster with 34% of these being endemic to the area (Schnabel et al., 2023). Technological advancements have allowed scientists to better study the deep sea and its organisms, and squat lobsters have been a useful model in studying depth transitions in relation to species distributions (Rodríguez-Flores et al., 2019, 2020).

Squat lobsters have a large and diverse number of symbiotic relationships that often influence their life history and ecology. Many of these symbioses are partnerships with other invertebrates such as sponges, corals, and sulfur-oxidizing microbes (Baeza, 2011). While squat lobsters are known to have mutualistic or commensal symbionts, over 80% of squat lobster symbionts are parasitic; these include rhizocephalans, nematomorphs, platyhelminth worms, and parasitic isopods (Boyko & Williams, 2011). These parasites can either partially (e.g., bopyrids) or completely (e.g., rhizocephalans) castrate their hosts (Boyko & Williams, 2011).

The majority of described squat lobster parasites (~70%) are members of the isopod suborder Epicaridea (Boyko & Williams, 2011). Epicaridea is divided into two super-families: Bopyroidea Rafinesque, 1815 and Cryptoniscoidea Kossmann, 1880, all species of which parasitize other crustaceans. Bopyroidea is further split into three families: Bopyridae Rafinesque, 1815, Entoniscidae Kossmann, 1880, and Ionidae H. Milne Edwards, 1840 (Boyko et al., 2013), of which only bopyrids (all ectoparasites typically found in the branchial chambers of hosts) are known from squat lobsters. The only other epicaridean found in squat lobster hosts is *Entophilus omnitectus* Richardson, 1903 (Cryptoniscoidea: Entophilidae), an internal parasite (Adkison & Collard, 1990; Boyko & Williams, 2015). Bopyridae is split into ten sub-families including Pseudioninae Codreanu, 1967, which contains the most common parasites of squat lobsters and was formerly considered the most primitive subfamily (e.g., Markham, 1986) until the genus *Pleurocryptella* Bonnier, 1900 was removed into its own subfamily, Pleurocryptellinae Williams & Boyko, 2024, now considered the basal subfamily within Bopyridae (Williams et al., 2024). Pseudioninae is the most diverse subfamily (~245 species) and, as with Pleurocryptellinae, contains species that settle as a cryptoniscus larva in the branchial chamber of their host and as the female isopod matures it creates a noticeable bulge in the carapace (Williams & Boyko, 2012). This difference between hosts with mature parasites allows researchers to easily identify and collect infested hosts, including squat lobsters. Based on the presence of this bulge, parasitization of squat lobsters has been documented back to the Late Jurassic period, with up to 10.4% of fossils in some areas showing evidence of parasitization by bopyrid isopods (Robins & Klompmaker, 2019).

Through their life cycle, almost all bopyrids parasitize two different crustacean hosts (Williams & Boyko, 2012): an intermediate copepod host and a definitive decapod host. The first larval stage, the epicaridium, leaves the female isopod’s marsupium and swims in search of a copepod host. Upon contact, the epicaridium larva will metamorphose into the microniscus stage which feeds on the hemolymph of the copepod. The microniscus will metamorphose into the cryptoniscus larva and leaves the copepod in search of the definitive host. Once a definitive host is found, the cryptoniscus larva settles onto it and matures into the juvenile state, known as a bopyridium. The bopyridium then travels to its final attachment site on the host. As the isopod grows in the branchial chamber, its body shape distorts resulting in mature females appearing more or less asymmetrical (Baba, 2005). The need for two different hosts means that the biogeography of bopyrids is influenced by more than just a single host species’ distribution.

Bopyrids are relatively well studied in some geographic regions (e.g., western Europe, see Bourdon, 1968; north-west Pacific coast of the United States, see Williams & Boyko, 2012), but many other areas, such as New Zealand, are understudied, especially for deeper water hosts (see Pike, 1961; Page, 1985). Due to the geographic isolation of New Zealand, many marine groups exhibit high endemicity to the area with crustaceans having an estimated average of 37% endemicity (Kelly et al., 2023; Costello, 2024).

The New Zealand Institute for Earth Science Ltd. (formerly the National Institute of Water and Atmospheric Research of New Zealand, NIWA) has a large series of squat lobster samples infested with bopyrids obtained from expeditions spanning from the 1940s to the present day. Among these samples, hosts from three genera (*Curtonida* Macpherson & Baba, *Phylladiorhynchus* Baba and *Scolonida* Macpherson & Baba) (see Machordom et al., 2022) yielded 38 parasitized specimens each bearing a pair of bopyrids (rarely, only a female) identified as belonging to *Parapleurocryptella* Bourdon, 1972. Prior to the present study, there were only two described species within *Parapleurocryptella*: *P. elasmonoti* Bourdon, 1972 parasitizing *Munidopsis squamosa* (A. Milne-Edwards) from Martinique (Caribbean Sea) and *P*. *minuta* Bourdon, 1972 parasitizing *Uroptychus gracilimanus* (Henderson) from Sumatra (Indonesia); both are known only from their type localities (Bourdon, 1972). Of the thirteen species of bopyrids known from New Zealand, none belong to *Parapleurocryptella* (Page, 1985), making this the first time the genus has been found in the region.

The purpose of this study was to examine the morphology of adult female, male, and larval features of the present specimens and to describe them as belonging to a new species. Additionally, we addressed some taxonomic issues within Pseudioninae and Pleurocryptellinae, including transfer of three genera to the latter subfamily.

## Materials and methods

Specimens stored in 70% ethanol were borrowed from the Earth Sciences New Zealand National Invertebrate Collection (NIC; prefix NIWA in material examined); all hosts were identified to species where possible. Sex (based on the presence or absence of gonopores on the third (female) and fifth (male) pereopods) of hosts was determined and carapace length (CL) was recorded using calipers and measured from the groove of the rostrum to the end of the cephalothorax (cf. Baba et al., 2009). In order to extract the parasites, the exoskeleton of the host was pulled back in the branchial region. Upon removal, the presence of female and/or male parasites was recorded, and they were measured using calipers or an ocular micrometer as total length (TL) from the anterior end of head to the posterior end of body. Several samples had the parasite(s) previously removed from the host, but the same measurements were recorded. The marsupium of bopyrid females was examined for the presence of eggs or larvae.

Males and epicaridium larvae were prepared for scanning electron microscopy (SEM) using 30 µm microporous specimen capsules (Electron Microscopy Services, EMS). Specimens were dehydrated using an ascending ethanol series (70% to 100%; 10 min for 70–95% and 15 min three times for 100%). After transfer from the final 100% ethanol dehydration, they were placed in a Samdri-795 sample dryer for critical point drying. The dried specimens were mounted on aluminum stubs and coated with gold using an EMS 550 sputter coater. Mounted specimens were examined using a FEI Quanta 250 SEM. Hosts and parasites were also photographed using a Macropod Pro kit; 10–20 images were aligned and stacked into a single composite image using Zerene Stacker software (http//zerenesystems.com/). Line drawings of males and females were completed using Olympus microscopes with drawing tube attachments and traced with a Wacom tablet. The straight-line tool on ImageJ was used to determine male and female maximum length, head length, and head width. Map figure was created using ArcGIS® software by Environmental Systems Research Institute (Esri). In order to avoid confusion, *Phylladiorhynchus* is abbreviated as *Ph*. throughout.

The holotype of *Pseudione kossmanni* was formerly in the collections of the Zoological Museum of Amsterdam (ZMA); now in Naturalis (RMNH) in Leiden.

## Results

**Fig. 1.**
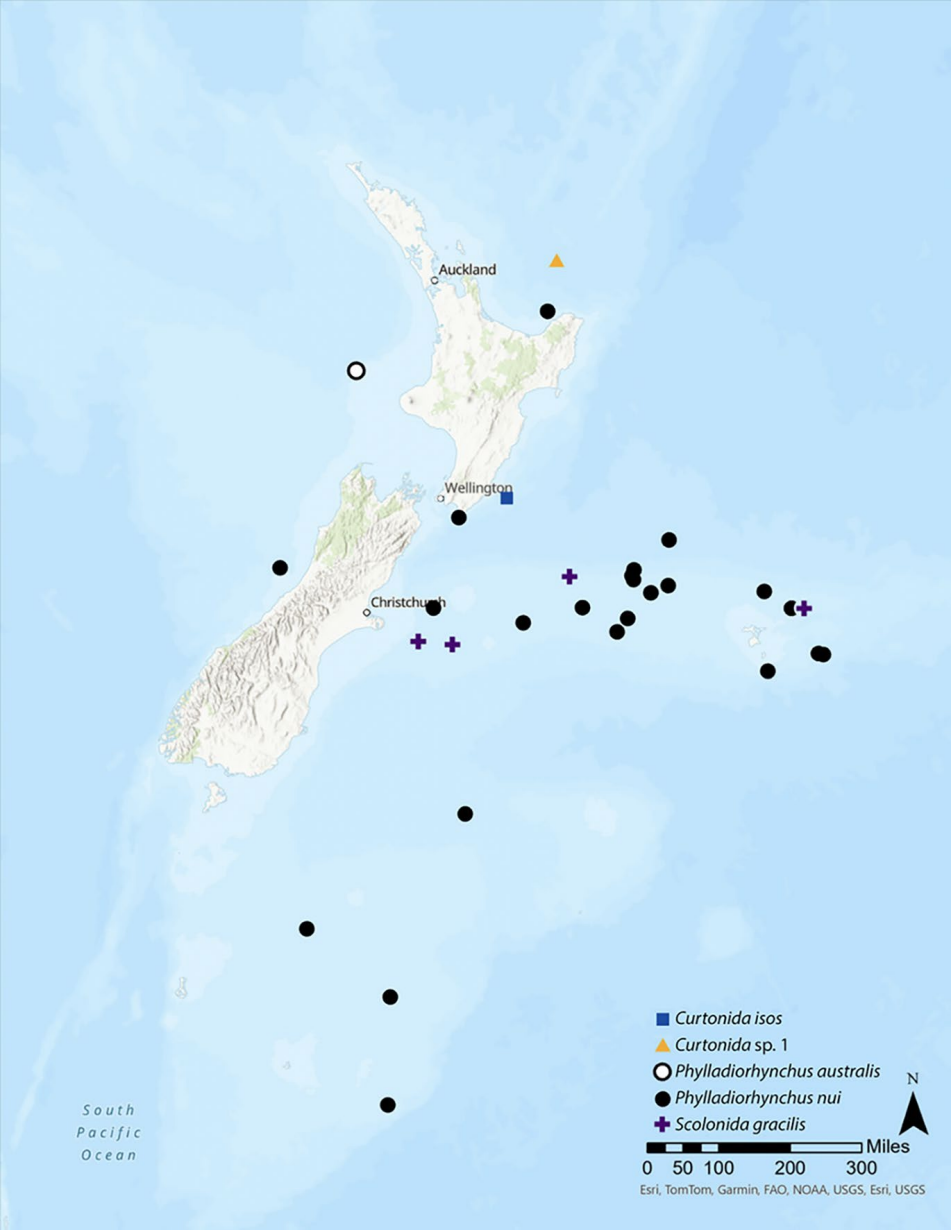
Geographic distribution of *Parapleurocryptella poha* **n. sp.** by host species: *Curtonida isos* (Ahyong & Poore), *Curtonida* sp. 1, *Phylladiorhynchus australis* Schnabel & Ahyong, *Phylladiorhynchus nui* Schnabel & Ahyong (type host), and *Scolonida gracilis* (Henderson).

In total, the new species of *Parapleurocryptella* was found on 38 hosts representing at least four species: *Curtonida isos* (Ahyong & Poore) (1 infested), *Curtonida* sp. 1 (1 infested; this host may be a new species of *Curtonida* but is in need of further study; Schnabel, pers. obs.), *Phylladiorhynchus australis* Schnabel & Ahyong (1 infested), *Phylladiorhynchus nui* Schnabel & Ahyong (31 infested), and *Scolonida gracilis* (Henderson) (4 infested). Among our samples, 32 of the 38 (84.2%) identified host squat lobsters belonged to the genus *Phylladiorhynchus* with *Ph. nui* being the most common host (Fig. 1). Schnabel & Ahyong (2019) reported a prevalence of bopyrids of approximately 9% (36/400) of *Ph. nui* sampled; most of these records (31) were the new species of *Parapleurocryptella*; thus, the prevalence of this species is ~7.8%.

**Fig. 2.**
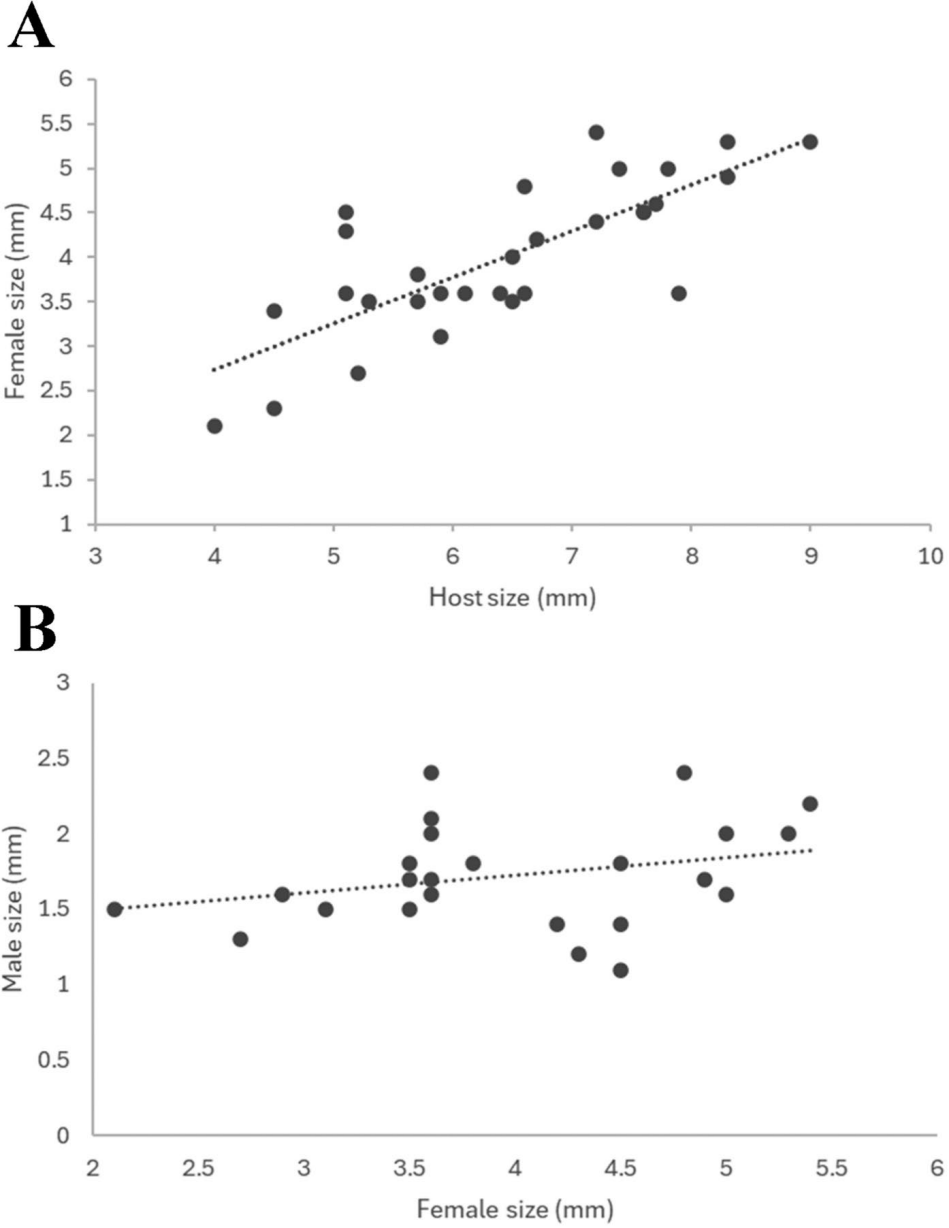
A, Relationship between female total body length of *Parapleurocryptella poha* **n. sp.** and carapace length of its host *Phylladiorhynchus nui* Schnabel & Ahyong (y = 0.52x + 0.658; R^2^ = 0.61; n = 30; P < 0.0001). B, Relationship between male total body length and female total body length of *P*. *poha* **n. sp.** (y = 0.116x + 1.26; R^2^ = 0.081; n = 22; P < 0.01).

Average female parasite length was 4.3 ± 1.5 mm (n=38) and average male parasite length was 1.8 ± 0.6 mm (n=30). The size of female parasites was significantly positively correlated with host length for *Ph. nui* (t=6.49, df = 28, p < 0.0001) (Fig. 2A). Specimens of the new species of *Parapleurocryptella* were found approximately equally in the right (54%; 20 of 37) and left (46%; 17 of 37) branchial chambers of hosts. As is typical, all mature females from the right gill chamber were dextral, and all females from the left gill chamber were sinistral; in one case an immature specimen was very slightly sinistral but found in the right branchial chamber. Male bopyrid length was positively correlated with female bopyrid length but this correlation was not significant (t=1.39, df = 20, p = 0.178) (Fig. 2B).

Two specimens of the new *Parapleurocryptella* species (NIWA 175097, NIWA 160810) were hyperparasitized by the rhizocephalan *Duplorbis koru* Kokasko, Williams, Wong & Chan, 2025 (Kokasko et al., 2025). One host specimen (*Ph*. *australis*) was doubly infested by the new species of *Parapleurocryptella* and a cryptoniscoid on the abdomen (cf. *Danalia* sp.), to be described in a separate publication (Williams et al., in prep).

### Systematics

Order Isopoda Latreille, 1816

Suborder Epicaridea Latreille, 1825

Family Bopyridae Rafinesque, 1815

Subfamily Pleurocryptellinae Williams & Boyko *in* Williams, Boyko & Stewart, 2024

Type genus.— *Pleurocryptella* Bonnier, 1900.

Other included genera.— *Paragigantione* Barnard, 1920, *Parapleurocryptella* Bourdon 1972, *Pagurocryptella* Boyko & Williams, 2010, *Pleurocryptina* Nierstrasz & Brender à Brandis, 1929.

Diagnosis.— **Female** body elongate or ovate, weakly distorted to distorted; head bilobed or not bilobed; frontal lamina present. Eyes present or absent. Maxilliped with setose articulated and segmented palp, or setose non-articulated palp, or without palp. Barbula with one or two long smooth lobes or one long lobe and one small nub on each side or one short smooth lobe on each side, median region smooth. Five or seven pairs of oostegites; oostegite 1 with ovate posterior lobe, smaller, or subequal to, or larger than anterior lobe; internal ridge smooth. Coxal plates and dorsolateral bosses present, tergal projections present or absent. Mediodorsal lobes absent. Pereopods not elongate; without propodal sockets. Pleon narrower or not narrower than pereon. Lateral plates absent or reduced; four or five pairs of smooth biramous pleopods or four pairs of smooth biramous pleopods and one pair of smooth uniramous pleopods or three pairs of biramous and two pairs of smooth uniramous pleopods, pleopods of one or two segments; uropods uniramous, smooth, distally bilobed or entire. **Male** approximately two to four times as long as wide, head narrower or as wide as pereon, pereomeres narrower or not narrower posteriorly. Eyes present or absent. Maxillipeds segmented, distally setose (in some species the maxilliped segmentation may be indistinct). Pereopods 1 and 2 larger than other pairs or all pereopods subequal. Midventral tubercles present or absent. Pleon of six pleomeres, five pairs of pleopods; posterolateral corners of pleomere 6 rounded or indented; uropods present, articulated or non-articulated. **Epicaridium larva** body tear-dropped shape with large yolk sac extending between uropods.

Remarks.— Pleurocryptellinae was erected by Williams et al. (2024) to contain the genus *Pleurocryptella* whose females had seven pairs of oostegites, an ovate posterior lobe on oostegite 1, males with maxillipeds of two segments, and epicaridium larvae with large posterior yolk sacs. Subsequently, Boyko et al. (2024) discovered that the epicaridium larvae of *Paragigantione* spp. also had posterior yolk sacs and noted that females have an ovate posterior lobe on oostegite 1 and males have maxillipeds of two segments but that females had only five pairs of oostegites. In large part due to the synapomorphy of the larval yolk sacs, *Paragigantione* was transferred from Pseudioninae to Pleurocryptellinae and the diagnosis of the subfamily was modified accordingly. Based on the data reported herein, we likewise transfer *Parapleurocryptella* from Pseudioninae to Pleurocryptellinae, as it shows the same set of diagnostic characters as *Paragigantione* that warranted placement in this subfamily.

There are two other genera that show characters suggesting placement in Pleurocryptellinae: *Pleurocryptina* Nierstrasz & Brender à Brandis, 1929 (one species) and *Pagurocryptella* Boyko & Williams, 2010 (two species) (Nierstrasz & Brender à Brandis, 1929; Boyko & Williams, 2010). Females of species of *Pagurocryptella* have seven pairs of oostegites, an ovate posterior lobe on oostegite 1 and the males have maxillipeds of two segments but larvae are unknown. Williams et al. (2024) suggested that, based on the unusual, albeit not unique, character of seven pairs of oostegites, *Pagurocryptella* might belong in Pleurocryptellinae but did not transfer it there; upon reconsideration, we now do so. *Pleurocryptina*, on the other hand, was not previously considered to belong to Pleurocryptellinae, but the female of the sole species has five pairs of oostegites, an ovate posterior lobe on oostegite 1, and males have maxillipeds of two segments; despite the lack of knowledge on the epicaridium larvae, we conclude that this genus is better placed in Pleurocryptellinae than Pseudioninae. Examination by CBB of the holotype of *Pseudione kossmanni* Nierstrasz and Brender à Brandis, 1923 (ZMA IS 100.549; RMNH) shows that this species belongs to *Pleurocryptella* as *Pleurocryptella kossmanni* (Nierstrasz and Brender à Brandis, 1923) **nov. comb.** Although the holotype is damaged, the female has subequal lobes of the first oostegite and rudimentary oostegites on pereomeres 6 and 7; the oostegite on pereomere 7 was previously reported by Nierstrasz and Brender à Brandis (1923). Additionally, and not reported by Nierstrasz and Brender à Brandis (1923), there was a large (ca. 2.5 mm) ovigerous *Cabirops* sp. female in the marsupium of the holotype (now loose in the jar).

## **Genus*Parapleurocryptella*Bourdon**, 1972

Type species.— *Parapleurocryptella minuta* Bourdon, 1972, by original designation.

Other included species.— *Parapleurocryptella elasmonoti* Bourdon, 1972, *Parapleurocryptella poha* **n. sp.**

Emended diagnosis.— Female ovate or elongate, body weakly to moderately distorted (≤~20%); head bilobed or not bilobed; frontal lamina present. Eyes absent. Maxilliped with setose articulated and segmented palp. Barbula with one short smooth lobe on each side, median region smooth. Five pairs of oostegites; oostegite 1 with ovate posterior lobe, smaller or larger than anterior lobe; internal ridge smooth. Coxal plates and dorsolateral bosses present, tergal projections absent. Mediodorsal lobes absent. Pereopods not elongate; without propodal sockets. Pleon narrower or not narrower than pereon. Lateral plates absent; five pairs of smooth biramous pleopods; uropods uniramous, smooth. **Male** approximately two times as long as wide, head narrower than pereon, pereomeres not narrower posteriorly. Eyes absent. Maxilliped segmented. Pereopods subequal in size. Midventral tubercles present or absent. Pleon of six pleomeres; five pairs of sessile pleopods; posterolateral corners of pleomere 6 rounded; uropods present.

Remarks.— The generic diagnosis is herein modified to accommodate the characters of the new species: females with a greater angle of asymmetry (~20°) than the previously described two species, and a bilobed head (not bilobed in other species). The males of the new species have midventral tubercles on pleomeres 1–4 that are absent in the type species (males of the other previously described species are unknown).

**Fig. 3.**
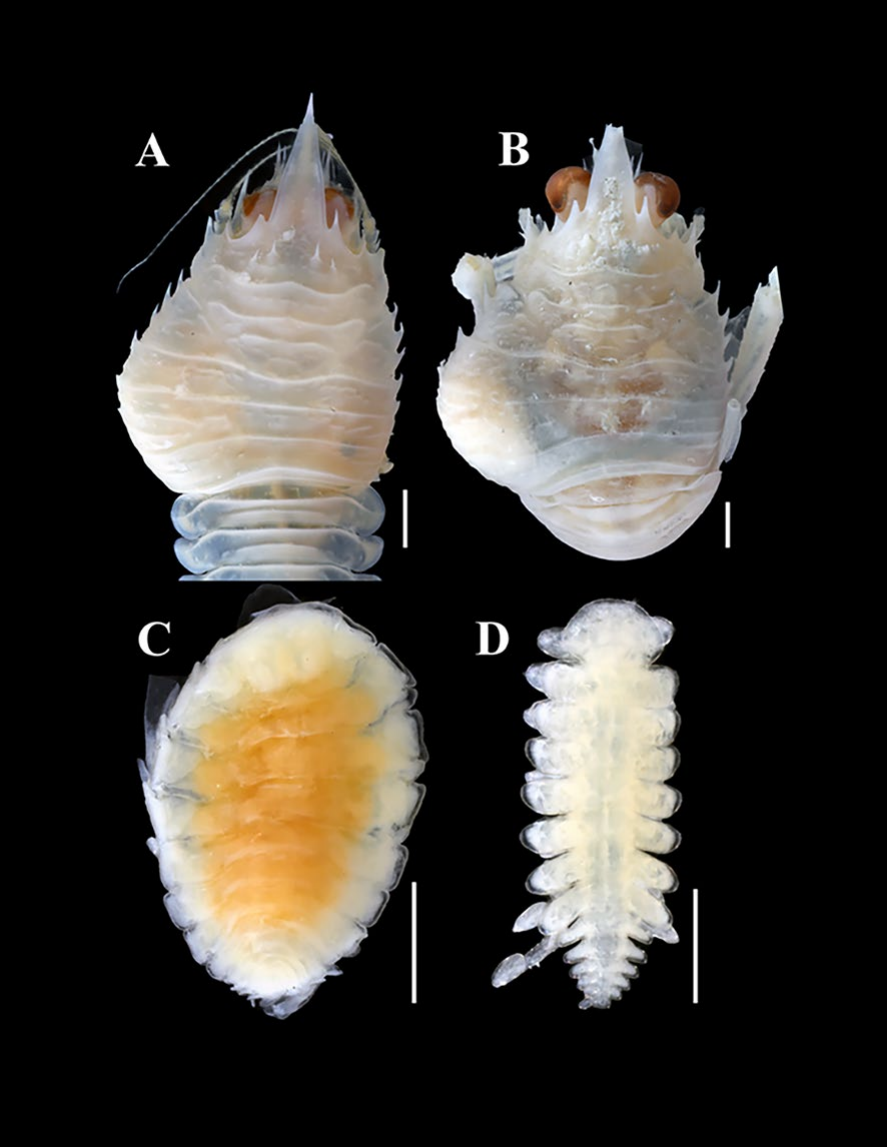
A, *Phylladiorhynchus nui* Schnabel & Ahyong, dorsal view of host (NIWA 21172) with swollen left branchial chamber containing *Parapleurocryptella poha* **n. sp.** B, *Ph*. *nui* dorsal view of host (NIWA 33667) with swollen left branchial chamber containing *P*. *poha* **n. sp.** C, Dorsal view of holotype female *P*. *poha* **n. sp.** (NIWA 182752) D, Dorsal view of allotype male *P*. *poha* **n. sp.** (NIWA 182753). *Scale-bars:* A–C = 1 mm; D = 0.5 mm.

**Fig. 4.**
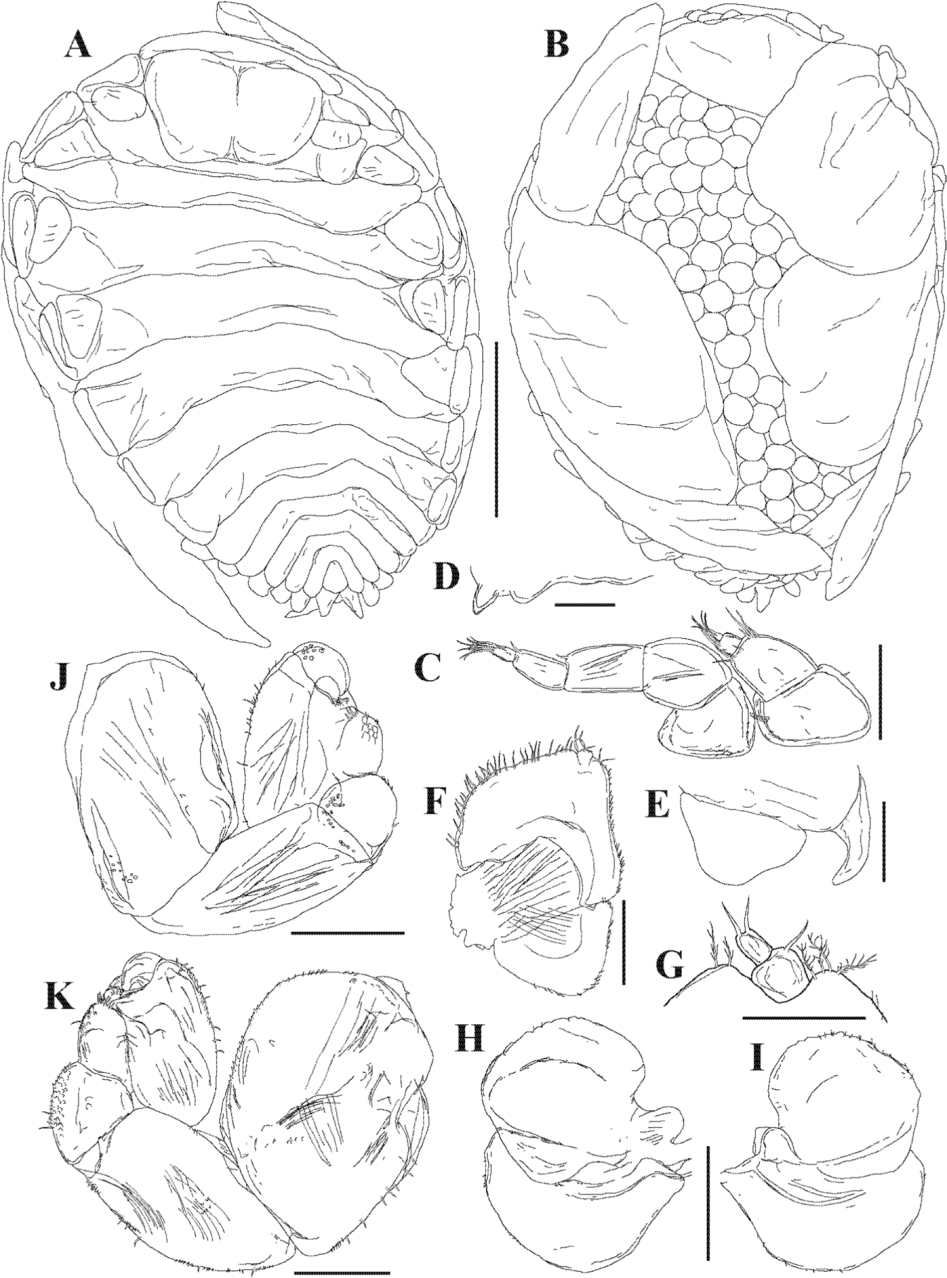
*Parapleurocryptella poha* **n. sp.** Female specimen (NIWA 175952) (A–D, F–K), female paratype (NIWA 182730) (E). A, Dorsal view; B, Ventral view; C, Left antennae; D, Right side of barbula; E, Left outer lobe of barbula and posterior lobe of maxilliped; F, Right maxilliped; G, Maxilliped palp; H, Left oostegite 1, inner view; I, Left oostegite 1, outer view; J, Pereopod 1; K, Pereopod 7. *Scale-bars:* A, B = 1 mm; C, G = 100 µm; D–F = 250 µm; H–K = 500 µm.

**Fig. 5.**
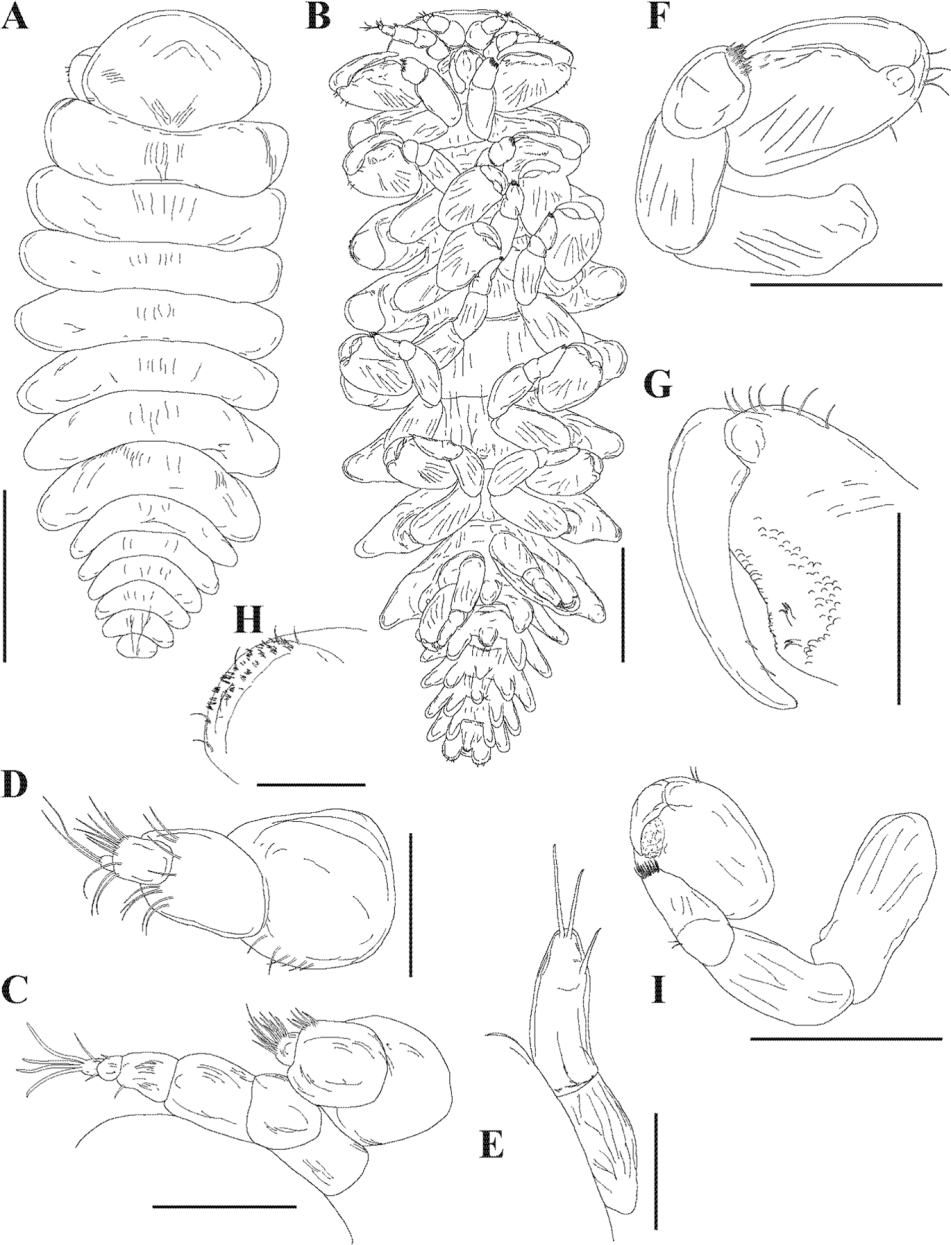
*Parapleurocryptella poha* **n. sp.** Male specimen (NIWA 175952) (A–D, F–I), male paratype (NIWA 182753) (E). A, Dorsal view (note: antennae omitted from this drawing); B, Ventral view; C, Antenna; D, Antennule; E, Maxilliped; F, Pereopod 1; G, Pereopod 1 detail; H, Side of pereomere 2; I, Pereopod 7. *Scale-bars:* A, B, 0.5 mm, C, D, F–I, 0.1 mm, E, 0.05 mm.

**Fig. 6.**
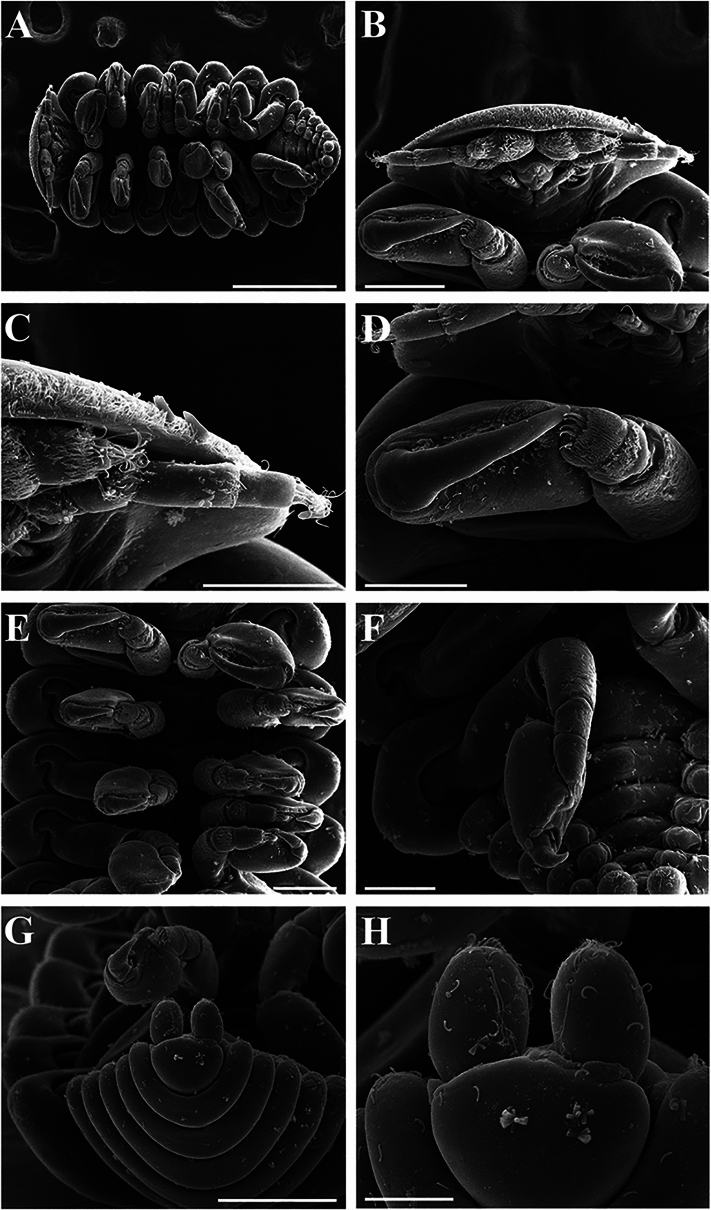
*Parapleurocryptella poha* **n. sp.** Paratype male (NIWA 182726). A, Ventral view; B, Head showing antennae, antennules and first pereopods; C, Close-up of antennae and antennules; D, Pereopod 1; E, Pereopods 1–5; F, Pereopod 7; G, Pleotelson and uropods, dorsal view; H, Close-up of uropods. *Scale-bars:* A, 500 µm, B, E, 150 µm, C, D, F, 100 µm, G, 200 µm, H, 50 µm.

**Fig. 7.**
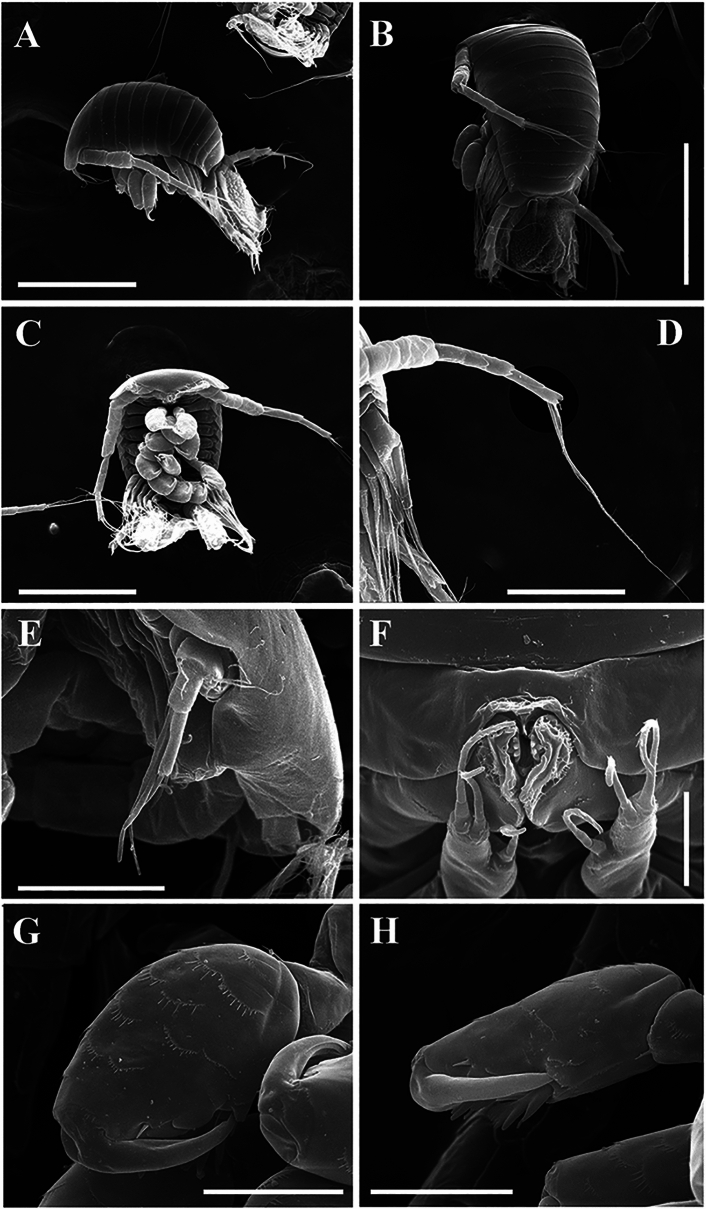
*Parapleurocryptella poha* **n. sp.** Epicaridium larva (NIWA 182726). A, Lateral view; B, Dorsal view; C, Ventral view; D, Antennae; E, Antennule; F, Maxillipeds and oral cone; G, Pereopod 1; H, Pereopod 6. *Scale-bars:* A–C, 200 µm, D, 100 µm, E, 50 µm, F, 10 µm, G, H, 30 µm.

**Fig. 8.**
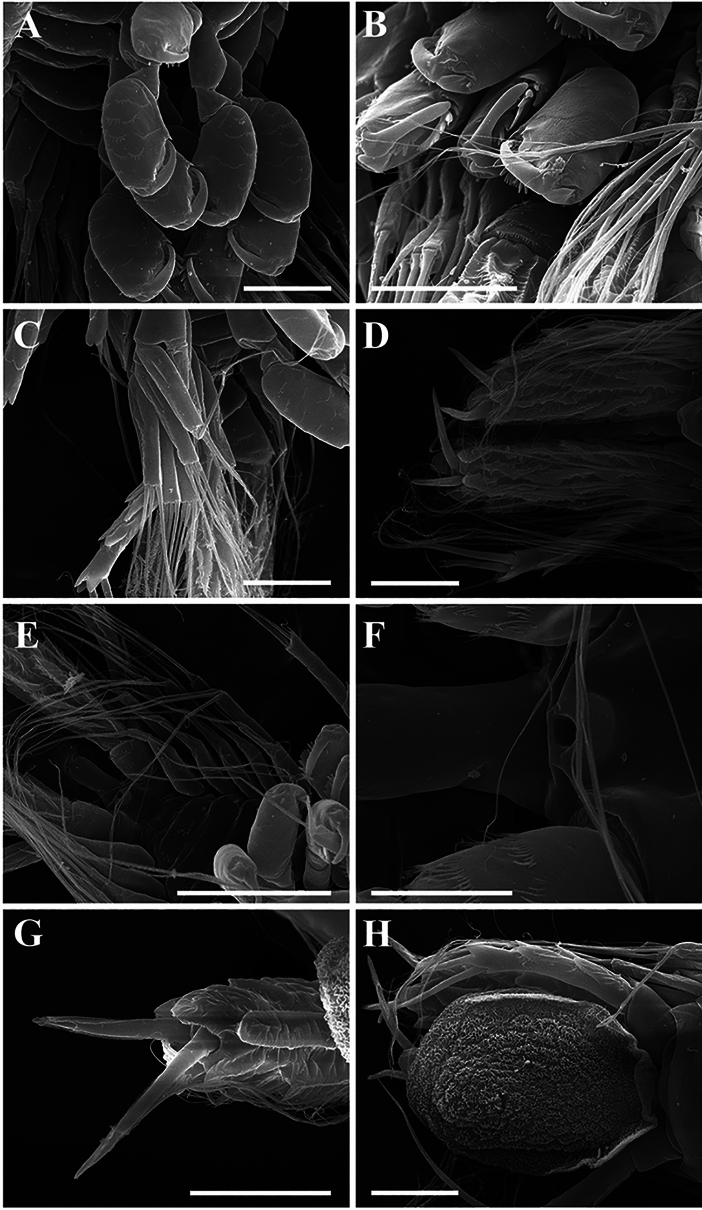
*Parapleurocryptella poha* **n. sp.** epicaridium larva (NIWA 182726). A, Pereopods overview; B, Pereopods 5, 6; C, Pleopods; D, Uropods and posterior end; E, Pleopods; F, Pore; G, Uropod; H, Yolk sac. *Scale-bars:* A–D, H, 50 µm, E, 100 µm, F, 20 µm, G, 40 µm.

***Parapleurocryptella poha *n. sp.** (Figs. 3–8)

*Pleurocryptella infecta*.—Page, 1985: 196 (in part; see remarks) (not *Pleurocryptella infecta* Nierstrasz & Brender à Brandis, 1923).

“bopyrid” Schnabel & Ahyong, 2019: 309 (in part), 326 (in part)

*Parapleurocryptella* sp. Kokasko et al. (2025): 5–10, Table 1 [specimens examined herein].

Type material examined.— Infesting *Phylladiorhynchus nui* Schnabel & Ahyong: Ovigerous female **holotype** (3.2 mm TL; NIWA 182752) and mature male **allotype** (1.8 mm TL; NIWA 182753), from right branchial chamber of male host (5.1 mm CL; NIWA 11606), Sta. V480, 41.2925°S, 176.5502°E, 725 m, New Zealand, coll. Hikurangi Giljanes voyage, 7 June 1994. **Paratypes**: Ovigerous female (4.5 mm TL) and mature male (1.4 mm TL), from left branchial chamber of male host (7.6 mm CL; NIWA 82442), Sta. TAN1206/53, 37.504°–37.501°S, 177.619°–177.614°E, 696–689 m, New Zealand, 20 April 2012 (NIWA 175960). Mature female (3.6 mm TL) (no male), from left branchial chamber of female host (5.9 mm CL; NIWA 21220), Sta. NZOI E572, 41.678°S, 175.257°E, 618 m, Hikurangi Margin, New Zealand, 30 March 1967 (NIWA 175946). Mature female (3.8 mm TL) and mature male (1.8 mm TL), from right branchial chamber of male host (5.7 mm CL; NIWA 21199), Sta. NZOI S894, 42.517°S, 170.335°E, 530 m, New Zealand, 19 July 1991 (NIWA 175945). Ovigerous female (3.6 mm TL) and mature male (2.4 mm TL), from right branchial chamber of male host (6.6 mm CL; NIWA 43332), Sta. TAN0104/148, 42.714–42.7095°S, 179.9585–179.9575°W, 893–980 m, Chatham Rise, New Zealand, 18 April 2001 (NIWA 175957). Ovigerous female (2.7 mm TL) and mature male (1.3 mm Tl), from female host (5.2 mm CL; NIWA 21272), Sta. V369, 42.8308°S, 179.9925°E, 1048 m, Chatham Rise, New Zealand, 11 Sept 1989 (NIWA 175949). Mature female (3.6 mm TL) and mature male (1.7 mm TL), from left branchial chamber of male host (6.4 mm CL; NIWA 24566), Sta. TAN1208/35, 42.911°–42.916°S, 179.962°–179.961°W, 751–758 m New Zealand, 19 June 2012 (NIWA 175950). Ovigerous female (4.5 mm TL) and mature male (1.1 mm TL) from right branchial chamber of female host (5.1 mm CL; NIWA 33680), Sta. TAN0705/155, 42.99883–43.000733°S, 176.34830–176.34870°W, 648–666 m, Chatham Rise, New Zealand, coll. Ocean Survey 2020, 16 April 2007 (NIWA 175956). Mature female (3.4 mm TL) with epicaridium larvae (no male), from left branchial chamber of female host (4.5 mm CL; NIWA 21243), Sta. V370, 42°4.70'S, 179°.0342'W, 1024 m, North Canterbury Region and Chatham Rise, New Zealand, 12 Sept 1989 (NIWA 175947). Ovigerous female (3.6 mm TL) and mature male (2.0 mm TL), from left branchial chamber of female host (7.9 mm CL; NIWA 21267 A), Sta. V371, 43.002°S, 179.000°W, 546 m, North Canterbury Region and Chatham Rise, New Zealand, 12 Sept 1989 (NIWA 182725 A). Ovigerous female (4.5 mm TL) and mature male (1.8 mm TL), from right branchial chamber of male host (7.6 mm CL; NIWA 24569), Sta. TAN1208/38, 43.165°–43.166°S, 179.468°–179.461°W, 522–523 m, North Canterbury and Chatham Rise, New Zealand, 19 June 2012 (NIWA 175951). Mature female (ca. 4.3 mm TL, damaged) and mature male (1.2 mm TL) from right branchial chamber of female host (5.1 mm CL; NIWA 33676), Sta. TAN0705/138, 43.2908°–43.2930°S, 175.5623°–175.5667°W, 640–644 m, Chatham Rise, New Zealand, 14 April 2007 (NIWA 175954). Ovigerous female (4.9 mm TL) and mature male (1.7 mm TL), from right branchial chamber of female host (8.3 mm CL; NIWA 21185), Sta. NZOI E43, 43.5°S, 174.5°E, 556 m, North Canterbury Region and Chatham Rise, New Zealand, 18 October 1965 (NIWA 182750). Mature female (2.3 mm TL) (no male), from left branchial chamber of male host (4.5 mm CL; NIWA 21172), Sta. NZOI V361, 43.506°S, 178.648°E, 340 m, North Canterbury Region and Chatham Rise, New Zealand, 6 September 1989 (NIWA 182749). Mature female (5.0 mm TL) with epicaridium larvae (~20 larvae on 2 SEM stubs) and mature male (2.0 mm TL) (on SEM stub), from left branchial chamber of male host (7.8 mm CL; NIWA 160814), Z6022, 43.70°S, 179.9167°E, 512 m, Chatham Rise, New Zealand, 24 January 1954 (NIWA 182726). Mature female (2.1 mm TL) and mature male (1.5 mm TL), from right branchial chamber of male host (4.0 mm CL; NIWA 21191), Sta. NZOI V387, 43.827°S, 176.997°E, 497–498 m, North Canterbury Region and Chatham Rise, New Zealand, 16 Sept 1989 (NIWA 182751). Ovigerous female (3.5 mm TL) and mature male (1.7 mm TL), from left branchial chamber of male host (5.7 mm CL; NIWA 33667), Sta. TAN0705/84, 43.9799°–43.9840°S, 179.6317°–179.6243°E, 531–532 m, Chatham Rise, New Zealand, 9 May 2007 (NIWA 175953). Mature female (4.0 mm TL) (no male), from right branchial chamber of female host (6.5 mm CL; NIWA 53605 B), Sta. TAN0905/97, 44.147°–44.148°S, 174.69–174.695°W, 440–600 m, Diamond Head Seamount, New Zealand, 26 June 2009 (NIWA 160660 B). Mature female (4.6 mm TL) (no male), from left branchial chamber of female host (7.7 mm CL; NIWA 53605 C), Sta. TAN0905/97, 44.147°–44.148°S, 174.69°–174.695°W, 440–600 m, Diamond Head Seamount, New Zealand, 26 June 2009 (NIWA 160660 C). Mature female (4.2 mm TL) with epicaridium larvae and mature male (1.4 mm TL), from left branchial chamber of female host (6.7 mm CL; NIWA 53605), Sta. TAN0905/97, 44.147°–44.148°S, 174.69°–174.695°W, 440–600 m, Diamond Head Seamount, New Zealand, 26 June 2009 (NIWA 160808). Mature female (4.4 mm TL) with epicaridium larvae (no male), from right branchial chamber of female host (7.2 mm CL; NIWA 53956) Sta. TAN0905/111, 44.148°–44.15°S, 174.691°–174.694°W, 458–648 m, Diamond Head Peak C, Andes Seamounts, New Zealand, 27 June 2009 (NIWA 160811). Ovigerous female (5.3 mm TL) and mature male (2.0 mm TL), from right branchial chamber of female host (8.3 mm CL; NIWA 53773 A), Sta. TAN0905/103, 44.158°S, 174.555°–174.559°W, 520–650 m, Iceberg Seamount, New Zealand (NIWA 182727 A). Ovigerous female (3.5 mm TL) and mature male (1.5 mm TL), from left branchial chamber of male host (6.5 mm CL; NIWA 53773 B), Sta. TAN0905/103, 44.158°S, 174.555°–174.559°W, 520–650 m, Iceberg Seamount, New Zealand (NIWA 182727 B). Mature female (4.8 mm TL) and mature male (2.4 mm TL), from right branchial chamber of male host (6.6 mm CL; NIWA 160815 A), Sta. Z6056, 44.5971°S, 176.067°W, 604 m, southeast of Pitt Island, Chatham Island, New Zealand, 3 February 1954 (NIWA 182728 A). Mature female (3.5 mm TL) with epicaridium larvae and mature male (1.8 mm TL), from right branchial chamber of female host (5.3 mm CL; 160815 B), Sta. Z6056, 44.5971°S, 176.067°W, 604 m, southeast of Pitt Island, Chatham Island, New Zealand, 3 February 1954 (NIWA 182728 B). Ovigerous female (3.1 mm TL) and mature male (1.5 mm TL), from left branchial chamber of female host (5.9 mm CL; 160815 C), Sta. Z6056, 44.5971°S, 176.067°W, 604 m, southeast of Pitt Island, Chatham Island, New Zealand, 3 February 1954 (NIWA 182728 C). Mature female (2.9 mm TL) with three externae of *Duplorbis koru* (NIWA 175096) in marsupium and two mature males (1.6 mm, 1.3 mm TL), from left branchial chamber of male host (two males, 4.7, 5.0 mm TL, both with swollen empty left branchial chambers, parasites loose in vial so host specimen not identifiable; NIWA ), Sta. NZOI E752, 47.687°S, 175.257°E, 618 m, New Zealand, 30 March 1967 (NIWA 175097). Ovigerous female (5.0 mm TL) and mature male (1.6 mm TL), from right branchial chamber of female host (7.4 mm CL; NIWA 21276), Sta. NZOI S16, 49.833°S, 170.223°E, 593 m, New Zealand, 14 Sept 1978 (NIWA 182729). Ovigerous female (5.4 mm TL) and mature male (2.2 mm TL), from left branchial chamber of female host (7.2 mm CL; NIWA 21162), Sta. NZOI F136, 51.333°S, 172.7°E, 547 m, Campbell Plateau, New Zealand, 30 January 1965 (NIWA 182730). Mature female (5.3 mm TL) with eight externae of *Duplorbis koru* (NIWA 175098) in marsupium (no male), from left branchial chamber of female host (9.0 mm CL; NIWA 21268), Sta. S48, 53.510°S, 172.400°E, 501 m, Campbell Plateau, New Zealand, 22 September 1978 (NIWA 160810).

Infesting *Phylladiorhynchus australis* Schnabel & Ahyong: **Paratypes**: Mature female (2.5 mm TL) and mature male (1.4 mm TL), from right branchial chamber of female host (3.6 mm CL; NIWA 21254), host also with a cryptoniscoid on abdomen (cf. *Danalia* sp.), NZOI Stn. E908, 38.633°S, 172.683°E, 256 m, 28 March 1968 (NIWA 175948).

Additional material examined.— Infesting *Curtonida isos* (Ahyong & Poore): Ovigerous female (3.9 mm TL) and mature male (3.6 mm TL), from left branchial chamber of male host (5.8 mm CL; NIWA 29281) New Zealand, Sta. TAN0616/67, 41.295-41.2942°S, 176.556-176.5610°E, 731-750 m, Hikurangi Rise, New Zealand, 10 November 2006 (NIWA 175952).

Infesting *Curtonida *sp. 1: Mature female with epicaridium larvae (5.5 mm TL) (no male), from right branchial chamber of male host (7.2 mm CL; NIWA 85217), Sta. TAN1206/99, 36.4453°–36.4428°S, 177.8392°–177.8403°E, 850-927 m, Clark Seamount, Kermadec Ridge, New Zealand, 24 April 2012 (NIWA 175961).

Infesting *Scolonida gracilis* (Henderson): Mature female (7.5 mm TL) and two mature male (2.3, 2.7 mm TL) infesting right branchial chamber of male host (12.9 mm CL; NIWA 78067), Sta. TAN1116/119, 42.8823°–42.8797°S, 178.2750°–178.27217°E, 578–585 m, Chatham Rise, New Zealand, 17 November 2011 (NIWA 175958). Ovigerous female (2.8 mm TL) and mature male (0.9 mm TL), from right branchial chamber of male host (4.5 mm CL; NIWA 33677), Sta. TAN0705/138, 43.2908°–43.2930°S, 175.5623°–175.5667°W, 638–644 m, Chatham Rise, New Zealand, 14 April 2007 (NIWA 175955). Ovigerous female (8.6 mm TL) and mature male (2.6 mm TL), from left branchial chamber of male host (16.6 mm CL; NIWA 78329), Sta. TAN1116/40, 44.166°–44.1683°S, 174.0428°–174.0455°E, 570 m, Chatham Rise, New Zealand, 7 November 2011 (NIWA 175959). Ovigerous female (9.1 mm TL) and mature male (2.5 mm TL), from left branchial chamber of male host (13.4 mm CL; NIWA 13476), Sta. NZOI E422, 44.25°S, 175.00°E, 615 m, New Zealand 15 October 1965 (NIWA 182747).

Type locality.— 41.2925°S, 176.5502°E, 725 m, New Zealand.

Distribution and depth ranges.— 36.4 to 53.5°S, 170.2°E to 174.56°W; 256 to 1048 m.

Hosts.— *Curtonida isos* (Ahyong & Poore), *Curtonida* sp. 1, *Phylladiorhynchus australis* Schnabel & Ahyong, *Ph*. *nui* Schnabel & Ahyong (type host), and *Scolonida gracilis* (Henderson).

Etymology.— The new species is named after the Polynesian word for a bag made from bull kelp to store food (Pōhā) and refers to the yolk sac of the epicaridium larvae. The new name is formed as a noun in apposition.

Description.— Female (Figs. 3, 4): Holotype (NIWA 182752) 3.2 mm long, maximal width 2.3 mm across pereomere 3, head length 0.6 mm, head width 0.8 mm. Body ovate (Fig. 4A, B), sinistrally rotated (angle of distortion ~20%). Head subrectangular in shape, partially bilobed (Figs. 3C, 4A); frontal lamina extending beyond anterior and lateral margins of head. Eyes absent. Barbula with one small to large smooth lateral lobe and one low rounded inner lobe; median region smooth (Fig. 4D, E). Antennules of three articles each, terminal article small, setose; antennae of five articles each, terminal article small, setose (Fig. 4C). Maxilliped (Fig. 4E, F) anterior lobe margin setose, segmented palp with complex setae at base (Fig. 4G), posterior lobe with rounded maxilliped spur (Fig. 4F). Five pairs of oostegites, marsupium incompletely closed, oostegite 1 (Fig. 4H, I) anterior lobe ovate, slightly larger than posterior rounded lobe, inner ridge smooth.

Pereon (Figs. 3C, 4A) of seven pereomeres, broadest across pereomere 3, pereomere 1 and 2 surrounding head, posterior borders of pereomeres 3–7 progressively more convex; dorsolateral bosses distinct on pereomeres 1–4, coxal plates on all pereomeres (Fig. 4E). Seven pairs of pereopods, subequal in size but differing in fusion of segments; pereopod 1 with propodus/carpus fusion (Fig. 4J), pereopod 7 with all segments distinct (hooked dactyl, ovate propodus, triangular carpus, triangular merus, quadrate ischium and large quadrate basis) (Fig. 4K).

Pleon (Figs. 3C, 4A) slightly narrower than terminal pereomere, six pleomeres including pleotelson (Fig. 4A). Pleomeres 1–5 each with pair of small, biramous pleopods; uropods uniramous, triangular, extending slightly past end of body (Fig. 4A).

Male (Figs. 3D, 5, 6): Allotype (NIWA 182753) 1.8 mm long, maximal width 0.7 mm across pereomere 3, head length 0.3 mm, head width 0.45 mm. Body (Fig. 5A, B) two times as long as wide at widest point of pereomere 3. Small setae sparsely spread over dorsal side of body (Fig. 5H). Head rounded, narrower than width of pereomere 1 (Figs. 5A, 6B). Antennae of six articles each (Figs. 5C, 6C); antennules of four articles each (Fig. 5C, D). Terminal article of antenna with four long setae, other smaller setae adjacent. All antennal segments setose, terminal segment with two long setae. Eyes absent. Maxilliped of two segments, terminal segment with three setae (Fig. 5E).

Pereon (Figs. 3D, 5A, B, 6A) of seven pereomeres. Pereomeres 1–4 all extended straight, 4–7 curved downward posterolaterally (Fig. 5A). Seven pairs of pereopods (Figs. 5B, 6A), subequal in size (Figs. 5B, 6E). Pereopod 1 with largest dactyl (Figs. 5F, 6D), size gradually decreasing posteriorly (Fig. 5F, I, 6E, F). Pereopods of six articles each, third and fourth segments setose. Carpus and propodus partly fused. Area surrounding dactyl textured and scaly (Fig. 5G).

Pleon (Figs. 3D, 5A, B, 6A, G) of six pleomeres, pleomeres directed posterolaterally. Midventral tubercles on pleomeres 1–4 and paired digitiform pleopods on pleomeres 1–5 (Fig. 5B). Uropods unramous, large, rounded (Figs. 5B, 6G, H), not visible in dorsal view when pleon curled inward (Fig. 5A); setose dorsally and terminally (Fig. 6H).

Epicaridium larva (Figs. 7, 8; NIWA 182726): length (including yolk sac) ~410 µm. Body of six pereomeres and six pleomeres (Fig. 7A–C). Antennules of three articles each, setae extending from segments 2 and 3 (Fig. 7E). Antennae of six articles each (four basal and two flagellar), terminal article with two long setae (Fig. 7D), antennae and terminal setae longer than body. Mouthparts with “toothed” mandibles (Fig. 7F). Maxilliped of two articles, distal article elongate with one medial stout seta and two distal stout setae, all with setules along length (Fig. 7F).

Six pereopods subequal in size. Pereopods with elongate dactyli and rounded propodi, propodus with scales and large plate-like setae with strongly serrate edges, closely applied near tip of dactylus (Figs. 7G, H, 8A, B). Five pairs of biramous pleopods, each exopod with three filamentous setae extending well beyond margins of body, each endopod bearing two long setae (Fig. 8C, E). Uropods large, biramous, endopods wider and longer than exopods, endopods with crenulations on surface and two stout terminal setae (Fig. 8D, G), exopods with smaller setae and scales along length plus one long and one shorter terminal seta (Figure 8D, H). Telson basal portion with pore (Fig. 8F), stalk-like telson extending into thin, broad, rounded yolk sac; yolk sac with textured cuticle and curled edge (Fig. 8H).

Remarks.**—** The two other species of *Parapleurocryptella*, *P. elasmonoti* and *P. minuta*, show several differences from *Parapleurocryptella poha* **n. sp.** Females of the new species have a greater angle of asymmetry (~20°), whereas *P. minuta* and *P. elasmonoti* have angles less than 10°. Females of *P*. *poha* **n. sp.** have a partially bilobed head, whereas those of *P. minuta* and *P. elasmonoti* do not.

The male of *P*. *elasmonoti* is unknown. *Parapleurocryptella minuta* males have antennules of three articles compared to the four seen in *P. poha* **n. sp.**, and antennae of five articles compared to six in *P. poha* **n. sp.** Males of *P. minuta* have isomorphic pereopods, whereas those of *P*. *poha* **n. sp.** have longer dactyli on the first pereopods and dactyli diminishing in size to from pereopod 1 to 7. The males of the new species have midventral tubercles on pleomeres 1–4, a trait that has not been documented within this genus before.

This is the first description of epicaridium larvae for any species in the genus *Parapleurocryptella*. Posterior external yolk sacs have been observed in only two other bopyrid genera: *Pleurocryptella* (e.g., Kato et al. 2022; Williams et al., 2024) and *Paragigantione* (Boyko et al., 2024). In addition to the presence of the yolk sacs, the epicaridium larvae of *Parapleurocryptella* *poha* **n. sp.** have well developed maxillipeds (segmented and with large terminal setae), a feature also found in species of *Pleurocryptella* and *Paragigantione*.

Page (1985) reported on material he identified as *Pleurocryptella infecta* from four New Zealand stations. It is not clear to which lot the figured specimens belong (Page, 1985: fig. 7) but that male and female are correctly identified as *P*. *infecta* whereas the female from NZOI Sta. E752 (herein NIWA 175097) is actually *Parapleurocryptella poha* **n. sp.** This specimen was from the squat lobster *Ph. nui* and was hyperparasitized by *D. koru* (Kokasko et al., 2025). The identity of the other specimens Page (1985) examined is not known.

Five of the 17 (~18.5%) female host specimens of *Ph. nui* parasitized by *P*. *poha* **n. sp.** had eggs, indicating that this parasite does not completely shut down host reproduction.

## Discussion

*Parapleurocryptella poha* **n. sp.** is the third species described in the genus *Parapleurocryptella*. The new species differs from both *P. minuta* and *P. elasmonoti* in morphology of males and females as well as in geographic distribution and host use. *Parapleurocryptella minuta* was reported infesting the chirostylid *Uroptychus gracilimanus* (Henderson) in Sumatra from 750 m and *P. elasmonoti* infests the galatheoid *Munidopsis squamosa* (A. Milne-Edwards) in Martinique from 191 fathoms (= 166.4 m) (A. Milne-Edwards & Bouvier, 1897). While these parasites have different geographic ranges, they are all found on deep water species of squat lobsters. *Parapleurocryptella poha* **n. sp.** is found widely distributed in New Zealand waters, parasitizing at least four host species at depths up to 1048 m (Fig. 1).

Examples of bopyrid species parasitizing hosts in different genera of squat lobsters are uncommon but have been previously reported (e.g., González & Acuña, 2004) and *P*. *poha* **n. sp.** is found on hosts belonging to two families of galatheoids (Munididae and Galatheidae, both in Galatheoidea). It has been suggested that the eco-physiological similarities of host groups such as squat lobsters may play an important role in host/parasite associations (i.e., there may be extensive host switching in the evolutionary history of these parasites; Boyko & Williams, 2009). Future studies need to address this question with accurate host and parasite identifications, phylogenies and co-evolutionary analyses.

Morphologically the new species possesses characters that place it in *Parapleurocryptella* but the generic diagnosis requires a slight revision in that females have a larger angle of distortion and a partially bilobed head than that seen in the previously described species. In addition, males of the new species have midventral tubercles on the first four pleomeres, which has not previously been documented in the type species. The three included species appear to form a well-defined group, although the males of *P. elasmonoti* remain to be described and epicaridium and cryptoniscus larvae need to be studied for the two previously described species.

A significant positive correlation was found between the size of the female bopyrids and host specimens, similar to findings in several other bopyrid/host relationships (e.g., Beck, 1980; Torres Jordá & Roccatagliata, 2002; Petrić et al., 2010; Varisco & Vinuesa, 2011; Romero-Rodríguez & Roman-Contreras, 2013; Cericola & Williams, 2015; Williams et al., 2019; Varisco et al., 2024). In addition, *P. poha* **n. sp.** likely reduces reproductive capability of hosts, as found in other bopyrids, including those on squat lobsters (see Boyko & Williams, 2011; Varisco & Vinuesa, 2011). However, over 18% of the female *Ph. nui* hosts parasitized by *P. poha* **n. sp.** had eggs, showing it is not a complete castrator. In contrast, the bopyrid *Pleurocrypta* sp. was reported to fully suppress oogenesis in the squat lobster *Iridonida speciosa* (von Martens) (Petrić et al., 2010; as *Munida rutllanti* Zariquiey Álvarez, a junior synonym).

The epicaridium larvae of *P. poha* **n. sp.** possess several notable characters including a well-developed maxilliped and presence of a yolk sac. These structures have been documented in only three species of *Pleurocryptella* (Kato et al., 2022; Williams et al., 2024) and two species of *Paragigantione* (Boyko et al., 2024); all three of these genera are now placed in Pleurocryptellinae. In the present study, the yolk sacs were observed with light microscopy and found to contain yolk granules. Typically, yolk is distributed in the gut of larvae, but it appears that in species of *Parapleurocryptella, Pleurocryptella,* and *Paragigantione* the yolk, or at least a portion of it, migrates posteriorly into the yolk sac. Compared to the larvae previously studied (Kato et al., 2022; Boyko et al., 2024; Williams et al., 2024) the yolk sac present in our specimens appears more deflated and flattened.

The presence of this yolk sac also calls into question aspects of this species’ life cycle. In most other bopyrid species, the epicaridium larvae hatch and enter the water column to find an intermediate copepod host, where they molt into microniscus larvae and feed (Williams & Boyko, 2012). The life cycle is continued when the larvae molt to the cryptoniscus stage, move off the copepod host and find and settle upon a definitive host. It is generally thought that the first larva that settles upon a host will develop into a female, whereas those that settle on the host afterwards will develop into males (Williams & Boyko, 2012). While this is assumed to be the life cycle for most bopyrid species (Williams et al., 2022), the allocation of yolk in epicaridium larvae of species in Pleurocryptellinae may indicate either a prolonged epicaridium stage, a bypassing of an intermediate host, or a potential advantage in maintaining position in the water column due to buoyancy.

Due to the uncertainty of the life cycle in terms of activity of the epicaridium larvae of the new species, future research is recommended to develop a complete life history of this bopyrid. If the presence of a yolk sac does not alter the need for an intermediate host, sampling copepods in the bopyrid’s range could yield infested hosts. Although this would be difficult, it could be aided by use of eDNA to determine locations where *P*. *poha* **n. sp.** is prevalent in the water column followed by extensive sampling of copepods (see Govindarajan et al., 2021 for similar studies utilizing eDNA in plankton studies). Rearing larvae in the lab from parasitized squat lobster hosts would be an ideal solution to testing larval biology behavior; however, due to the depths at which the host species are found (>500 m) this might not be feasible. Thus, determining key aspects of the natural history of this species, including the identity of the intermediate copepod species used as well as intermediate host specificity, will require novel approaches in future research.

## Data Availability

Type material is deposited in the National Institute of Water & Atmospheric Research Ltd (NIWA), Wellington, New Zealand. (see text for details) and is available for study.
